# EVIDENT Smartphone App, a New Method for the Dietary Record: Comparison With a Food Frequency Questionnaire

**DOI:** 10.2196/11463

**Published:** 2019-02-08

**Authors:** Jose I Recio-Rodriguez, Carmela Rodriguez-Martin, Jesus Gonzalez-Sanchez, Emiliano Rodriguez-Sanchez, Carme Martin-Borras, Vicente Martínez-Vizcaino, Maria Soledad Arietaleanizbeaskoa, Olga Magdalena-Gonzalez, Carmen Fernandez-Alonso, Jose A Maderuelo-Fernandez, Manuel A Gomez-Marcos, Luis Garcia-Ortiz, EVIDENT Investigators

**Affiliations:** 1 Primary Health Care Research Unit Institute of Biomedical Research of Salamanca La Alamedilla Health Center Salamanca Spain; 2 Health Service of Castilla y León Valladolid Spain; 3 Primary Care Prevention and Health Promotion Research Network Barcelona Spain; 4 Faculty of Health Sciences University of Burgos Burgos Spain; 5 Department of Nursing University of Extremadura, Plasencia Campus Salamanca Spain; 6 Department of Physical Activity and Sport Sciences Faculty of Psychology, Education and Sport Sciences Blanquerna Ramon Llull University Barcelona Spain; 7 Department of Physical Therapy Faculty of Health Sciences Blanquerna Ramon Llull University Barcelona Spain; 8 Center for Health and Social Research University of Castilla-La Mancha Cuenca Spain; 9 Primary Health Care Research Unit of Bizkaia Basque Health Service-Osakidetza Bilbao Spain; 10 Torre Ramona Health Center Aragon Health Service Zaragoza Spain; 11 Casa del Barco Health Center, Castilla and León Health Service Valladolid Spain; 12 Department of Medicine, University of Salamanca Salamanca Spain; 13 Department of Biomedical and Diagnostic Sciences, University of Salamanca Salamanca Spain; 14 redIAPP: Spanish Research Network for Preventive Activities and Health Promotion in Primary Care Salamanca Spain

**Keywords:** technology assessment, biomedical, telemedicine, energy intake, diet records, surveys and questionnaires

## Abstract

**Background:**

More alternatives are needed for recording people’s normal diet in different populations, especially adults or the elderly, as part of the investigation into the effects of nutrition on health.

**Objective:**

The aim of this study was to compare the estimated values of energy intake, macro- and micronutrient, and alcohol consumption gathered using the EVIDENT II smartphone app against the data estimated with a food frequency questionnaire (FFQ) in an adult population aged 18 to 70 years.

**Methods:**

We included 362 individuals (mean age 52 years, SD 12; 214/362, 59.1% women) who were part of the EVIDENT II study. The participants registered their food intake using the EVIDENT app during a period of 3 months and through an FFQ. Both methods estimate the average nutritional composition, including energy intake, macro- and micronutrients, and alcohol. Through the app, the values of the first week of food recording, the first month, and the entire 3-month period were estimated. The FFQ gathers data regarding the food intake of the year before the moment of interview.

**Results:**

The intraclass correlation for the estimation of energy intake with the FFQ and the app shows significant results, with the highest values returned when analyzing the app’s data for the full 3-month period (.304, 95% CI 0.144-0.434; *P*<.001). For this period, the correlation coefficient for energy intake is .233 (*P*<.001). The highest value corresponds to alcohol consumption and the lowest to the intake of polyunsaturated fatty acids (*r*=.676 and *r*=.155; *P*<.001), respectively. The estimation of daily intake of energy, macronutrients, and alcohol presents higher values in the FFQ compared with the EVIDENT app data. Considering the values recorded during the 3-month period, the FFQ for energy intake estimation (Kcal) was higher than that of the app (a difference of 408.7, 95% CI 322.7-494.8; *P*<.001). The same is true for the other macronutrients, with the exception g/day of saturated fatty acids (.4, 95% CI −1.2 to 2.0; *P*=.62).

**Conclusions:**

The EVIDENT app is significantly correlated to FFQ in the estimation of energy intake, macro- and micronutrients, and alcohol consumption. This correlation increases with longer app recording periods. The EVIDENT app can be a good alternative for recording food intake in the context of longitudinal or intervention studies.

**Trial Registration:**

ClinicalTrials.gov NCT02016014; http://clinicaltrials.gov/ct2/show/NCT02016014 (Archived by WebCite at http://www.webcitation.org/760i8EL8Q)

## Introduction

### Background

Within the framework of intervention studies, the analysis of current or recent dietary intake can be approached with a wide variety of methods. In general, these methods are based on either a record of actual food intake over several days or an estimation using tools that assess the frequency of consumption of different foods [1]. Food frequency questionnaires (FFQs) allow us to estimate normal food consumption habits over a relatively recent period (months or years) by recording the frequency and amount with which the foods included in a set list are eaten. This type of questionnaire is most frequently used in epidemiological studies because they are cheap, simple, fast, and allow individual intake to be estimated with just 1 administration. The main drawback is that they usually do not have complete data, their list of foods is finite, and they require specific information from the population of interest, including the main sources of nutrients in the normal diet of the zone in which the study is conducted [2,3].

The review by Illner et al [2] suggests that food data collected with cell phones could improve collection compared with using conventional records because of the possibility of recording in real time. In addition, higher intraobserver reliability has been observed in comparison with written formats. Estimates of dietary intake based on records made in periods of 3 to 7 days could reduce costs. However, validity still appears to be limited when estimating an individual’s intake [4], given the necessary prior training and increased effort involved in data processing.

Different apps have proven their validity against a conventional method such as 24-hour recall. The apps Easy diet diary, My meal mate, and Food now obtained good correlations and a nonsignificant difference for energy intake [5-7] and small differences of means for some food groups [8]. In addition, other apps such as My fitness PAL or e-DIA correlate positively regarding the consumption of certain food groups [8,9], although they may overestimate that of carbohydrates and lipids, especially in individuals with a higher energy intake [9]. All these apps, however, have been validated in a relatively young audience and little is known about the behavior of older smartphone users.

Research into the effect of food on health requires new tools and technologies to quantify food intake in a valid, flexible, and simple way [3]. However, more evidence is needed before recommending the app of these alternatives for recording the normal diet of different populations, especially among adults or the elderly [2]. In the EVIDENT II study [10], an app for the recording of dietary patterns was developed, which, in addition to promoting the adoption of healthy lifestyles by the user, can be useful for gathering data regarding food intake and their subsequent use in nutritional intervention studies.

### Objectives

The objective of this study was to compare the estimated values of energy consumption, macro- and micronutrients, and alcohol collected with the EVIDENT II smartphone app in a week, a month, and 3 months, with the data estimated by means of a frequency questionnaire of food consumption in an adult population aged between 18 and 70 years.

## Methods

### Study Design and Population

Participants were selected from among those included in the EVIDENT II clinical trial intervention group [10]. The objective of this study was to assess the effectiveness of adding the use of a smartphone app to brief lifestyle improvement counseling (physical activity and eating habits) in a sample of the adult population. Overall, 6 groups from the Red Española de Investigación para Actividades Preventivas y Promoción de la Salud en Atención Primaria (REDIAPP; Spanish Research Network for Preventive Activities and Health Promotion in Primary Care) participated in the study. Subjects aged between 18 and 70 years were included. Subjects were excluded if they were unable to do exercise or follow the Mediterranean diet, or if they met any of the exclusion criteria. These criteria were the known presence of coronary or cerebrovascular atherosclerotic disease; heart failure; moderate or severe chronic obstructive pulmonary disease; musculoskeletal disease involving limited walking; advanced respiratory, renal, or hepatic disease; severe mental disease; or a treated oncological disease diagnosed in the last 5 years [10]. Of the 415 subjects in the intervention group (brief counseling + smartphone app) of the EVIDENT II study [10], 53 did not use the app to record their diet; hence, for the study presented in this manuscript, 362 subjects were included. This sample size allows the detection of a correlation coefficient of .15 or higher between the energy intake estimated by the FFQ and that estimated with the app, accepting an alpha risk of .05 and a beta risk of .20 in a bilateral contrast.

### Variables and Measurement Instruments

#### Estimation of Average Daily Nutritional Composition With the EVIDENT App

The EVIDENT app is a smartphone app developed by the computer company CGB and the Grupo de Investigaciónen Atención Primaria de Castilla y León (Castilla y León Primary Care Research Group) of the REDIAPP (intellectual property registration number 00/2014/2207).

**Figure 1:**
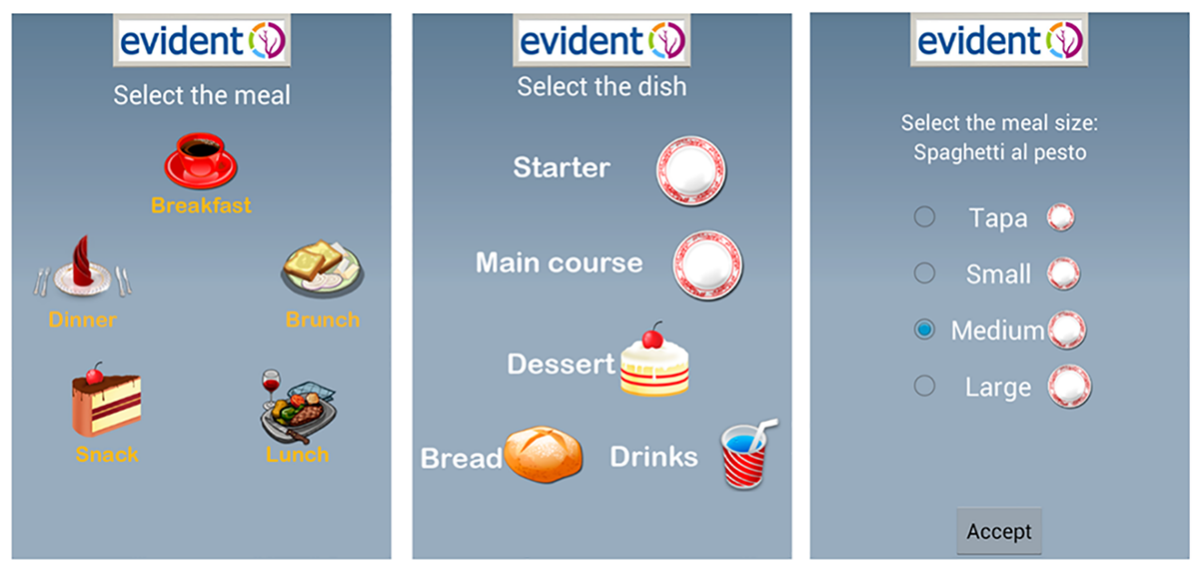
EVIDENT app main screen and selection of dishes.

**Figure 2:**
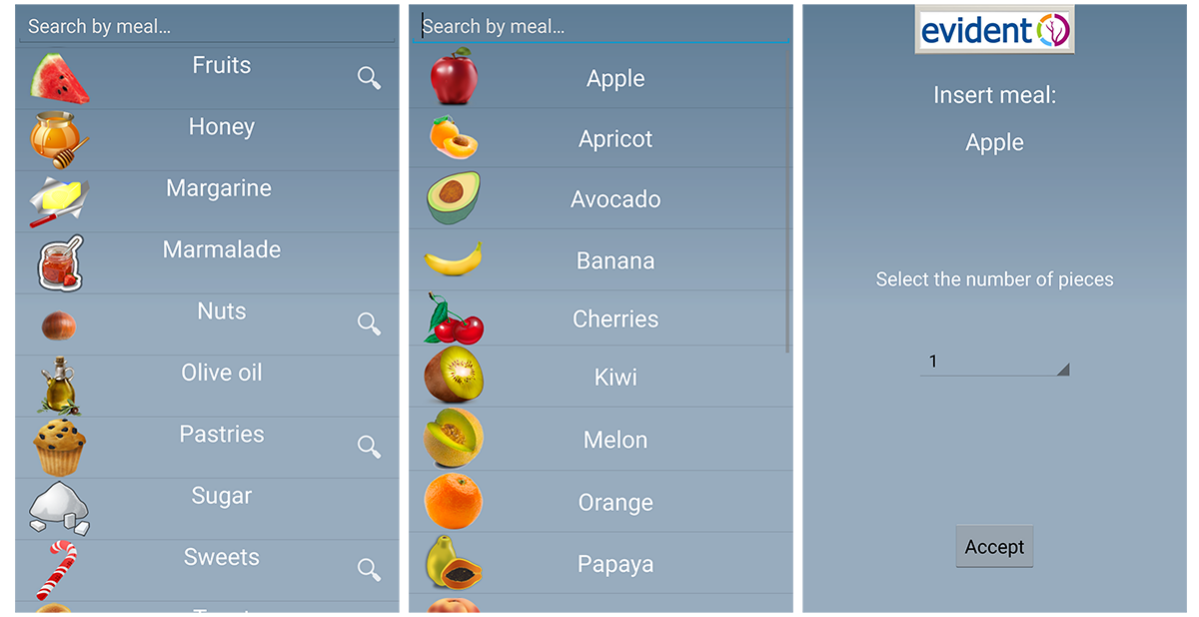
EVIDENT app food menu.

**Figure 3:**
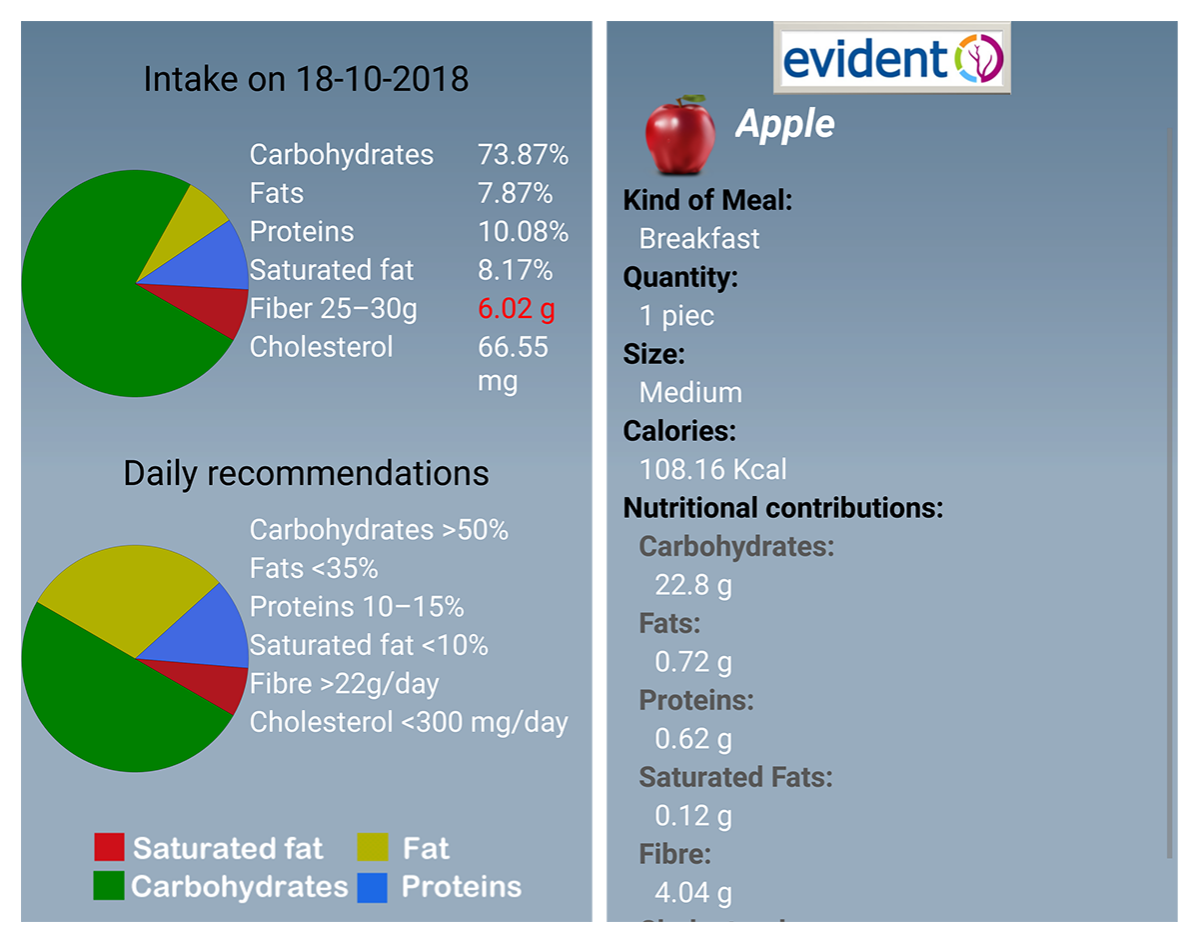
EVIDENT app feedback information.

The app allows the user to enter information regarding lifestyle habits such as diet. Within the app, foods are organized by groups that include dairy products, eggs, meat, fish, vegetables, fresh fruits, pulses, cereals, oils and fats, pastries, cakes and cookies, processed foods, snacks, beverages, bread, nuts, pasta, rice, salads, seafood, sauces, soups, and creams. The app was configured with the characteristics of each participant (age, sex, weight, and height). The subjects were required to enter their food intake daily (breakfast, midmorning snack, lunch, afternoon snack, and dinner) and select dishes and foods from the app menu (Figure 1). In the case of breakfast, midmorning, and afternoon snacks, the user selects each of the foods and their quantity listed on the app in the standard measures used by the reference population (Figure 2). For the lunch and dinner record, the type of dish and the size are selected (Figure 1). Other foods consumed at different times are recorded with the meal closest to them in time. On completing the daily record, the user is provided with 2 types of information by the app (Figure 3). The first covers the average nutritional composition of the corresponding day in terms of energy intake and macronutrients. This information was estimated using food composition tables and serving sizes provided by the user. The second concerns the nutritional composition of each ingested food corresponding to the portion size selected. In addition, each individual receives general weekly notifications with feedback messages about how well the general recommendations regarding certain aspects of the diet such as the consumption of fruits and vegetables or olive oil are being applied. This study presents the daily estimates of energy intake, consumption of macro- and micronutrients, and alcohol during the first week of recording, the first month, and the total time that individuals used the app (3 months). The average daily energy intake is expressed in Kcal, whereas the intake of macronutrients and alcohol is expressed in g/day. The micronutrients are expressed in terms of their respective units of consumption.

#### Estimation of Average Daily Nutritional Composition With the Food Frequency Questionnaire (FFQ)

A self-administered FFQ validated for the Spanish population was used [11]. This questionnaire covers 137 foods frequently used among the reference population, along with their standard portion sizes. These foods are organized into groups that include dairy products, eggs, meat, fish, vegetables, fresh fruits, pulses, cereals, oils and fats, pastries and cakes, processed foods, snacks, and beverages. Within each of these categories, a certain number of products are included. After receiving instructions from researchers, participants indicated the frequency with which each item was consumed during the last year on a scale of 9 response options (never or almost never, 1-3 times a month, once a week, 2-4 times a week, 5-6 times a week, once a day, 2-3 times a day, 4-6 times a day, or more than 6 times a day). The questionnaire data were used to estimate daily energy consumption expressed in Kcal and intake of macronutrients and alcohol expressed in g/day. Micronutrients were quantified in terms of their respective units of consumption. For this study, we used data obtained from the FFQ conducted for 3 months.

#### Other Measurements

Data on the sociodemographic characteristics of the population (age and sex); educational level and occupation; smoking and personal history of hypertension, dyslipidemia, and diabetes mellitus were collected.

Hypertension was considered present with figures of ≥140 mm Hg systolic blood pressure and/or ≥90 mm Hg diastolic blood pressure or if the subject was being treated with antihypertensive agents [12]. Dyslipidemia was determined with total cholesterol of ≥240 mg/dL or triglycerides of ≥200 mg/dL or lipid-lowering drug treatment [13]. For diabetes mellitus 2, we applied the American Diabetes Association criteria (glycated hemoglobin ≥6.5%, fasting plasma glucose ≥126 mg/dL, plasma glucose ≥200 mg/dL after 2 hours during an oral tolerance test of glucose, random plasma glucose ≥200 mg/dL, or the use of antidiabetic treatment) [14].

Smoking history was assessed through questions about the participant’s smoking status (smoker or nonsmoker). We considered smokers to be subjects who currently smoke or who stopped smoking less than 1 year ago.

#### Ethical Considerations

The study was approved by the clinical research ethics committee of the Salamanca health care area (June 21, 2013), and all participants gave written informed consent in accordance with the general recommendations of the Declaration of Helsinki [15].

#### Statistical Analysis

Descriptive statistics regarding clinical and sociodemographic characteristics of the studied population were expressed by means and SDs for the continuous variables and frequency distribution for the qualitative variables.

Concordance of the energy intake estimations produced by the app and the FFQ per week, month, and 3 months was analyzed using the intraclass correlation coefficient (ICC). In addition, the Bland-Altman method was used to calculate the limits of agreement between the 2 measurement tools. The comparison of means regarding energy consumption, macronutrients, and alcohol between the 2 measuring instruments was conducted with the *Student* *t* test. In addition, the Pearson correlation coefficient was used to analyze the relationship between the estimations of macro- and micronutrients and alcohol intake from the 2 measurement tools and between the 3 estimates of the app.

All analyses were performed with SPSS version 23.0 (IBM Corporation, Armonk, NY, USA) and an alpha risk of .05 was set as the limit of statistical significance.

## Results

### Characteristics of the Study Population

Our sample comprised 362 individuals with a mean age of 52 (SD 12) years (214/362, 59.1% women). Regarding cardiovascular risk factors, 35.9% (130/362) had a diagnosis of arterial hypertension, 28.5% (103/362) had dyslipidemia, 20.4% (74/362) were smokers, and 7.5% (27/362) had diabetes mellitus type 2 (Table 1). Among the 362 participants, 235 (235/362, 64.9%) registered their food intake more than 61 days (2 months) on the app, 62 (62/362, 17.1%) between 31 and 60 days (between 1 and 2 months), and 65 (65/362, 18.0%) less than 30 days (<1 month).

### Comparision Between Food Frequency Questionnaire and EVIDENT Smartphone App

**Figure 4:**
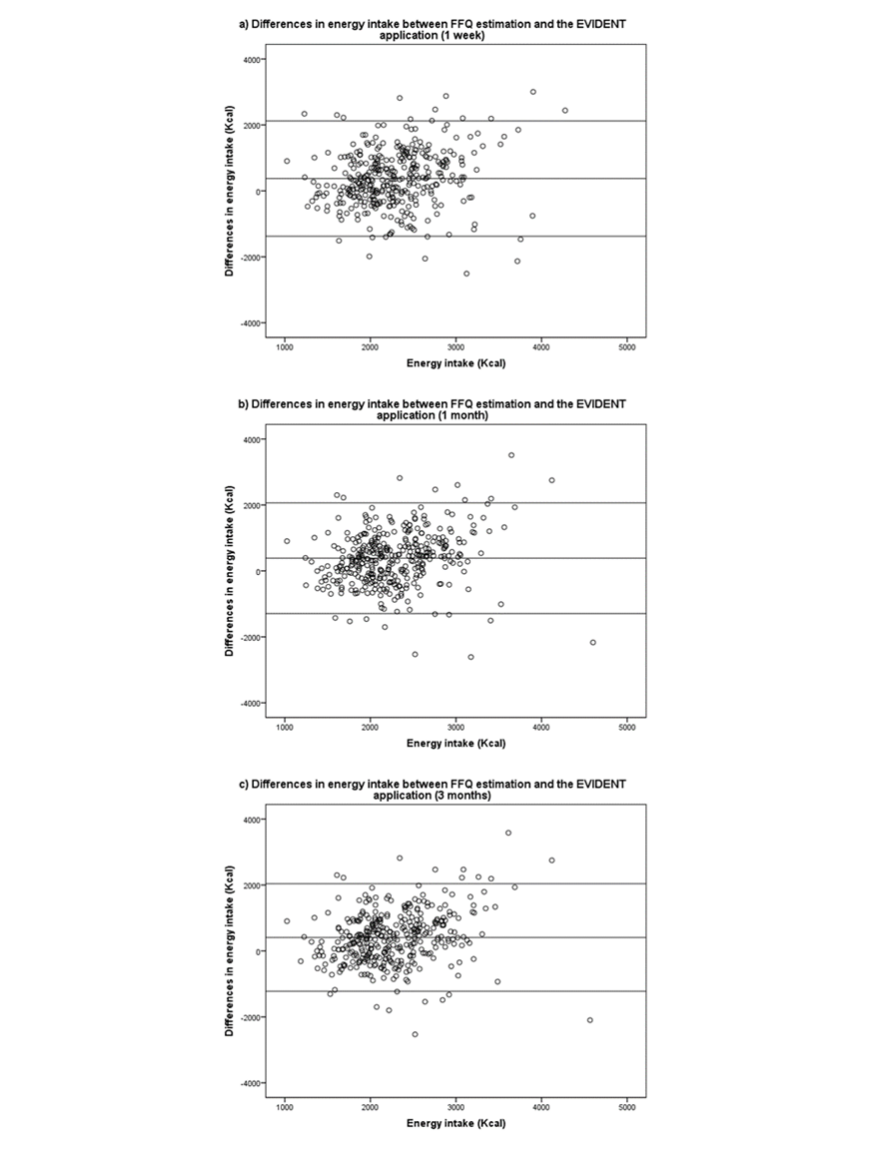
Bland-Altman Plots with the differences in energy intake estimation (Kcal) between the food frequency questionnaire (FFQ; 3 months) and data records of the application (1 week, 1 month and 3 months). (a) Limit of agreement for the estimation of the energy intake between FFQ and EVIDENT application (1 week): 374 Kcal (-1373 to 2121); (b) Limit of agreement for the estimation of the energy intake between FFQ and EVIDENT application (1 month): 386 Kcal (-1291 to 2063); (c) Limit of agreement for the estimation of the energy intake between FFQ and EVIDENT application (3 months): 408 Kcal (-1223 to 2040).

ICC between the FFQ and app for the estimation of energy intake at 1 week, 1 month, and 3 months shows a significant association in all 3 cases, with the highest ICC yielded by the 3-month record (.304, 95% CI 0.144-0.434; Table 2). The Bland-Altman plot was used to analyze the concordance of energy intake estimated by the FFQ at 3 months and the EVIDENT app at 1 week, 1 month, and 3 months (Figure 4), with a limit of agreement at 3 months of 408 Kcal (95% CI −1223 to 2040).

Figures for the estimation of daily energy, macronutrient, and alcohol intakes are higher with the FFQ than those provided by the EVIDENT app (Table 3). The lowest values for energy, macronutrients, and alcohol were found after 3 months of data recording. As can be seen, the value estimated through the FFQ for energy (Kcal) was higher than that yielded by the app, with a difference of 408.7 (95% CI 322.7-494.8; *P*<.001). This is repeated with the remaining macronutrients, with the exception of saturated fatty acids (g/day), in which a difference of 0.4 (95% CI −1.2 to 2.0; *P*=.62) was observed. The average values of energy, macronutrients, and alcohol estimates recorded with the app can be seen in Table 4.

**Table 1:** Baseline characteristics of the study population (N=362).

Baseline characteristics		Statistics^a^
Age (years), mean (SD)		52 (12)
Females, n (%)		214 (59.1)
**Work situation, n (%)**		
	Works outside of home	196 (54.1)
	Homemaker	48 (13.3)
	Retired	70 (19.3)
	Student	7 (1.9)
	Unemployed	41 (11.3)
**Educational level, n (%)**		
	University studies	102 (28.2)
	Middle or high school	184 (50.8)
	Elementary school	76 (21.0)
**Smoking, n (%)**		
	Nonsmoker	168 (46.4)
	Smoker	74 (20.4)
	Former smoker	120 (33.1)
**BMI^b^ categories, n (%)**		
	BMI <25	97 (26.8)
	BMI 25 to 30	152 (42.0)
	BMI >30	113 (31.2)
Hypertension, n (%)		130 (35.9)
Dyslipidemia, n (%)		103 (28.5)
Type 2 diabetes mellitus, n (%)		27 (7.5)

^a^Categorical variables are expressed as n (%) and continuous variables as mean (SD).

^b^BMI: body mass index.

**Table 2:** Intraclass correlation coefficient for energy intake.

Comparisons	Intraclass correlation (95% CI)	*P* value
FFQ^a^ and EVIDENT app: 1 week	.203 (0.021-0.352)	.02
FFQ and EVIDENT app: 1 month	.267 (0.099-0.404)	.01
FFQ and EVIDENT app: 3 months	.304 (0.144-0.434)	<.001
EVIDENT app (1 week, 1 month, and 3 months)	.941 (0.929-0.951)	<.001

^a^FFQ: food frequency questionnaire.

**Table 3:** Comparison of consumption of energy, macronutrients, and alcohol between the food frequency questionnaire and EVIDENT app.

Nutritional composition	FFQ^a^, mean (SD)	Mean difference of FFQ-EVIDENT app at 1 week (95% CI)	Mean difference of FFQ-EVIDENT app at 1 month (95% CI)	Mean difference of FFQ-EVIDENT app at 3 months (95% CI)
Energy intake (Kcal/day)	2467.3 (729.8)	374.0 (281.9 to 466.1)	386.2 (297.7 to 474.6)	408.7 (322.7 to 494.8)
Proteins (g/day)	106.9 (29.5)	20.2 (16.5 to 23.9)	20.7 (17.1 to 24.3)	21.5 (18.0 to 25.1)
Carbohydrates (g/day)	260.4 (88.1)	59.8 (49.6 to 70.0)	60.8 (50.9 to 70.7)	63.8 (54.1 to 73.4)
Fats (g/day)	103.8 (37.9)	9.0 (4.1 to 14.0)	9.8 (4.9 to 14.6)	10.5 (5.8 to 15.2)
Saturated fats (g/day)	30.2 (12.3)	0.1 (−1.5 to 1.8)^b^	0.1 (−1.5 to 1.8)^b^	0.4 (−1.2 to 2.0)^b^
Monounsaturated (g/day)	45.9 (17.6)	1.7 (−0.6 to 4.1)^b^	2.2 (0.0 to 4.3)^b^	2.5 (0.4 to 4.6)
Polyunsaturated (g/day)	16.9 (8.6)	4.4 (3.4 to 5.4)	4.4 (3.4 to 5.4)	4.5 (3.5 to 5.5)
Cholesterol (g/day)	458.8 (175.9)	125.5 (105.2 to 145.8)	127.2 (107.5 to 146.9)	130.2 (110.9 to 149.4)
Fiber (g/day)	27.5 (10.5)	1.9 (0.7 to 3.2)	1.9 (0.7 to 3.2)	2.5 (1.3 to 3.7)
Alcohol (g/day)	9.2 (13.3)	4.0 (2.9 to 5.2)	3.9 (2.8 to 5.0)	3.8 (2.7 to 5.0)

^a^FFQ: food frequency questionnaire.

^b^*P*>.05.

**Table 4:** Estimation of the average daily consumption of energy, macronutrients, and alcohol with data collected through the EVIDENT app during the first week, first month, and throughout the 3 months.

Nutritional composition	EVIDENT app 1 week, mean (SD)	EVIDENT app 1 month, mean (SD)	EVIDENT app 3 months, mean (SD)
Energy intake (Kcal/day)	2093.2 (602.5)	2081.1 (577.0)	2058.5 (557.9)
Carbohydrates (g/day)	86.7 (24.6)	86.2 (22.3)	85.4 (21.9)
Proteins (g/day)	200.6 (60.5)	199.6 (58.6)	196.6 (57.6)
Fats (g/day)	94.7 (34.6)	94.0 (33.1)	93.2 (31.7)
Saturated fats (g/day)	30.0 (13.9)	30.0 (13.9)	29.8 (13.2)
Monounsaturated fats (g/day)	44.2 (15.7)	43.7 (14.1)	43.4 (13.5)
Polyunsaturated fats (g/day)	12.5 (4.9)	12.5 (4.9)	12.4 (4.8)
Cholesterol (g/day)	333.3 (125.1)	331.6 (113.0)	328.6 (106.9)
Fiber (g/day)	25.5 (8.3)	25.5 (8.3)	25.0 (8.2)
Alcohol (g/day)	5.2 (7.7)	5.3 (7.6)	5.3 (7.6)

Table 5 shows the correlations between the EVIDENT app’s 1-week estimates for daily energy intake and the consumption of macronutrients and alcohol on the one hand, and the 1-month and 3-month records on the other. The values for energy correlate highly (*r*=.823 and *r*=.774; *P*<.001 all), although the highest correlations correspond to alcohol consumption (*r*=.894 and *r*=.865; *P*<.001), whereas the lowest correlations are those regarding cholesterol intake (*r*=.757 and *r*=.669; *P*<.001).

Table 6 shows the correlations between energy, macronutrient, and alcohol intakes recorded by the FFQ and the EVIDENT app for the first week, the first month, and at 3 months. The coefficients are higher when the FFQ is correlated with the 3-month data of the app. Energy intake yields a correlation coefficient of .233 (*P*<.001). The highest value corresponds to alcohol consumption and the lowest to the intake of polyunsaturated fatty acids (*r*=.676 and *r*=.155; *P*<.001), respectively.

Among the micronutrients (vitamins and minerals), we found significant correlations between FFQ and app estimates (Table 7). The highest concordance values were found for calcium and vitamin C intake (*r*=.316 and *r*=.319; *P*<.001), respectively, whereas the lowest correlations corresponded to phosphorus and vitamin D (*r*=.106 and *r*=.110; *P*<.05), respectively.

**Table 5:** Correlation of the consumption of nutrients between the intake estimated by the EVIDENT app in the first week, registration of the first month, and the record of the 3 months.

Nutritional composition	Correlation of the consumption between first week and first month	Correlation of the consumption between first week and three months
Energy intake (Kcal/day)	.823^a^	.774^a^
Carbohydrates (g/day)	.845^a^	.800^a^
Proteins (g/day)	.789^a^	.722^a^
Fats (g/day)	.800^a^	.731^a^
Saturated fats (g/day)	.825^a^	.763^a^
Monounsaturated fats (g/day)	.775^a^	.715^a^
Polyunsaturated fats (g/day)	.802^a^	.733^a^
Cholesterol (g/day)	.757^a^	.669^a^
Fiber (g/day)	.835^a^	.777^a^
Alcohol (g/day)	.894^a^	.865^a^

^a^*P*<*.* 01.

**Table 6:** Correlation of the nutrient consumption between the estimated values with the food frequency questionnaire and the registration through the EVIDENT app of the first week, first month, and 3 months.

Nutritional composition	Correlation between FFQ^a^ with first week records by the app	Correlation between FFQ with first month records by the app	Correlation between FFQ with 3 months records by the app
Energy intake (Kcal/day)	.135^b^	.202^c^	.233^c^
Carbohydrates (g/day)	.147^c^	.224^c^	.268^c^
Proteins (g/day)	.172^c^	.207^c^	.204^c^
Fats (g/day)	.165^c^	.220^c^	.232^c^
Saturated fats (g/day)	.276^c^	.291^c^	.311^c^
Monounsaturated fats (g/day)	.118^b^	.220^c^	.236^c^
Polyunsaturated fats (g/day)	.111^b^	.130^b^	.155^c^
Cholesterol (g/day)	.154^c^	.193^c^	.204^c^
Fiber (g/day)	.204^c^	.280^c^	.314^c^
Alcohol (g/day)	.647^c^	.675^c^	.676^c^

^a^FFQ: food frequency questionnaire.

^b^*P*<.05.

^c^*P*<.01.

**Table 7:** Correlation of mineral and vitamin consumption between the values estimated by the food frequency questionnaire (FFQ) and data collected through the registration of the EVIDENT app of the first week, first month, and 3 months.

Micronutrients		Correlation between FFQ^a^ with first week records by the app	Correlation between FFQ with first month records by the app	Correlation between FFQ with 3 months records by the app
**Minerals**				
	Calcium (mg/day)	.259^b^	.295^b^	.316^b^
	Iron (mg/day)	.190^b^	.254^b^	.259^b^
	Iodo (µg/day)	.155^b^	.178^b^	.198^b^
	Magnesium (mg/day)	.175^b^	.277^b^	.299^b^
	Zinc (mg/day)	.175^b^	.210^b^	.221^b^
	Selenium (µg/day)	.189^b^	.244^b^	.232^b^
	Phosphorus (mg/day)	.093	.109^c^	.106^c^
	Sodium (mg/day)	.124^c^	.157^b^	.151^b^
	Potassium (mg/day)	.197^b^	.267^b^	.278^b^
**Vitamins**				
	Vitamin A (mcg/day)	.141^b^	.167^b^	.166^b^
	Vitamin D (mcg/day)	.094	.092	.110^c^
	Vitamin C (mg/day)	.262^b^	.308^b^	.319^b^
	Vitamin B1 (mg/day)	.155^b^	.189^b^	.224^b^
	Vitamin B2 (mg/day)	.205^b^	.244^b^	.248^b^
	Niacin (mg/day)	.130^c^	.181^b^	.171^b^
	Vitamin B6 (mg/day)	.210^b^	.236^b^	.225^b^
	Folic acid (mcg/day)	.231^b^	.276^b^	.298^b^
	Vitamin B12 (mcg/day)	.139^b^	.196^b^	.196^b^
	Retinol (mcg/day)	.163^b^	.151^b^	.162^b^
	Carotenoids (mcg/day)	.230^b^	.275^b^	.274^b^

^a^FFQ: food frequency questionnaire.

^b^*P*<.01.

^c^*P*<.05.

## Discussion

### Principal Findings

Dietary records with the EVIDENT app yielded significant intraclass correlation with the FFQ in terms of energy intake. Similarly, the correlation between both instruments regarding the estimation of daily macro- and micronutrients intake and alcohol consumption was statistically significant. Furthermore, the correlations between the values obtained with the FFQ and with the EVIDENT app increase with longer app recording periods. The results of this study allow us to hypothesize that the use of electronic devices can be an alternative to FFQ for food recording in the context of longitudinal or intervention studies.

The increasing use of smartphones has produced an increase in the number of apps aimed at recording food intake. Many of these apps [6,7,16,17] have been compared against a traditional method of collecting information such as 24-hour recall with a young population. These apps have in common a high correlation for the estimation of energy intake, with small and insignificant differences between the app and 24-hour recall methods and with slightly higher values recorded by 24-hour recall for all measurements. The EVIDENT app’s estimates of energy intake are significantly lower than those estimated with the FFQ. The correlation between both instruments of measurement is significant and rises as the food recording period using the app increases. However, this correlation is low compared with other works [6,7,16,17]. There are several explanations for this finding. First of all, the EVIDENT app was compared with an FFQ rather than a 24-hour recall. This aspect is key to understanding the results. The 24-hour recall consists of collecting the most detailed information possible about the foods and beverages consumed in the preceding 24 hours. It is a method widely used in cross-sectional studies, but the great variety of available foods and ways of preparation makes it more difficult to estimate the rations consumed. In addition, one of the main limitations is that a single day of analysis does not reflect the usual pattern of consumption. When a comparison is made with a measuring instrument that explores food consumption over a short period (24 hours), a high correlation is expected as the food intake in that period is limited and the time gap between the records of both tools is closer, so there is less risk of memory bias. FFQs, meanwhile, estimate the nutritional intake over a longer in this case, 1 year. During this period, variability in foods is much higher and may be influenced by aspects such as seasonality or festivities that could alter food consumption. The FFQ seem to be a good instrument to estimate the nutritional composition over long periods (1 year or more) and the 24-hour recall in shorter periods (between 1 day and a week). However, for the estimation of energy and nutrient intake in the context of clinical investigations, it is necessary to use instruments capable of reporting beyond recent intake. The results of this study suggest that the use of the EVIDENT app can be a good measuring instrument for the estimation of nutritional composition in the context of longitudinal or intervention studies, although the implementation in clinical practice of this type of food registration tools could be difficult if there is not enough motivation to register them for long periods.

### Comparison With Prior Work

Among the macronutrients, the highest correlation is found for carbohydrates and fiber and the lowest, although significant, for polyunsaturated fatty acids. However, the correlation coefficients maintain similar values for all dietary components, which lend the app a high degree of cohesion. Again, the higher the number of days recorded, the greater the correlation. The estimation for energy intake and consumption of macronutrients show higher values when FFQ is used, which reinforces the conclusions of many other studies that indicate that FFQs may overestimate food consumption compared with other assessment instruments [18,19]. There are also notable differences in the process of data collection between the 2 tools for estimating the nutritional composition. Both the app and the FFQ collect the same types of food. However, the amount of products available for selection, within each category, is greater in the app. In this way, for example, in the selection of fish, the app has a greater range of products to select, distinguishing between each of them in a particular way. Although the FFQ tends to group more products into subcategories such as blue fish, white fish, or salted fish, not all fishes within the same subcategory have the same nutritional composition. In this sense, the app could be much finer in the estimation of macro- and micronutrients.

In the study conducted by Ashman et al [16], which also analyzed the results obtained with an app and a questionnaire for the estimation of micronutrient intake, moderate correlations (*r*=.47-.94; *P*<.05 in all of them) were found. However, among other information, the study subjects contributed characteristics regarding the preparation of the food consumed, which may have been influenced by subjective aspects linked to particular preferences in cooking as well as to certain customs. The population consisted of pregnant women, possibly under specific care and dietary care regimes. Moreover, previous research has shown that the 3 days, during which the app was used, would not be a sufficient period to accurately estimate micronutrient intake [20]. Regarding alcohol consumption, Ambrosini et al [6], in line with our results, also found a moderate correlation using an app and the 24-hour recall questionnaire. However, the study concluded that the app may have underestimated the consumption of alcohol compared with the 24-hour recall questionnaire. In addition, subjects only used the app for 4 days. A possible explanation of the difference between the different correlations found in the studies of Ashman and Ambrosini [6,16], for the consumption of macro- and micronutrients, and our study is that in the EVIDENT study, throughout the registration period, participants received daily notifications with recommendations to improve their dietary habits. These recommendations were part of the intervention of the EVIDENT trial and were aimed at achieving greater adherence to the Mediterranean diet and better adaptation to the recommendations for consuming macronutrients in the context of the usual diet. Another aspect that can explain the results are the different characteristics of the population studied in terms of age and health. The samples studied by Ashman [16] and Ambrosini [6] are relatively small (N=25 and N=50), with a young age average (29 and 31 years, respectively), and with a high percentage of women. However, the participants of the EVIDENT study are between 18 and 70 years of age, with an average of 52 years. In addition, the percentage of cardiovascular risk factors (hypertension, diabetes, dyslipidemia, and smoking) is slightly higher than in the general population. These circumstances highlight the heterogeneity of this sample and, therefore, the difficulty of finding more accurate correlations of nutrients between the different estimation instruments.

### Limitations

One of the main limitations of this study is the traditional instrument to which the app’s records were compared, an FFQ. This questionnaire was validated for the reference population (Spanish population) and uses the nutritional composition data of 137 normally consumed foods in Spain for the daily estimation. The data involved are those of the year before the interview. Other apps have used shorter periods (24 or 48 hours) and have compared their results against 24-hour recall. These studies have obtained better correlation results but these results were to be expected, given the very limited period during which intake data were collected.

### Conclusions

The EVIDENT app correlates significantly with FFQ in the estimation of energy intake, macro- and micronutrients, and alcohol consumption. This correlation grows as the app’s food recording period increases. The EVIDENT app can be a good alternative for gathering information on energy intake and the consumption of macronutrients, in the context of longitudinal or intervention studies.

## Abbreviations

BMI: body mass index
FFQ: food frequency questionnaire
ICC: intraclass correlation coefficients
REDIAPP: Red Española de Investigación para Actividades Preventivas y Promoción de la Salud en Atención Primaria [Spanish Research Network for Preventive Activities and Health Promotion in Primary Care]
